# Fibroid Tumors of the Uterus1Presidential address delivered before the annual meeting of the Buffalo Academy of Medicine, June 9, 1903.

**Published:** 1903-10

**Authors:** Herman E. Hayd

**Affiliations:** Buffalo, N. Y.


### BUFFALO MEDICAL JOURNAL.

VOL. XLIII.—LIX. OCTOBER, 1903. NO. 3.

### ORIGINAL COMMUNICATIONS.

### Fibroid Tumors of the Uterus.[1]

BY HERMAN E. HAYD, M. D., Buffalo, N. Y.

FIRST, permit me to express my thanks and my appreciation for the honor you conferred upon me, in electing me your presiding officer. Many of the best men in the profession of our city have filled this chair before me, and I assure you I shall always look back with pride and pleasure to this signal mark of your respect.

#### THE ACADEMY AND ITS WORK.

The year's work, which we have just completed, has been a very creditable one, and the character of the papers and the discussions would compare favorably with those of our national societies, which it has been my privilege often to attend. While we have reason for gratification at the constant accessions to our membership, still we cannot help but feel dissatisfied and disappointed that there are so many capable and otherwise eligible men in our city, who have not allied themselves with our Academy. The Academy of Medicine was formed for the promotion of the science and art of medicine, and to bring together for mutual intercourse and friendship the various members of the profession. How much it has accomplished, and how much it has failed to accomplish, each and all of us realize. However, we can be reasonably content in the thought that the Academy has grown to be a power in the community, and that it stands for all that is good and best in medicine. Its good opinion and endorsement are asked for by public sentiment, and great questions which affect the well-being of the community must bear the stamp of the Academy's approval. Our early days were a

1. Presidential address delivered before the annual meeting of the Buffalo Academy of Medicine, June 9, 1903.

little stormy, and some of the strong bulwarks of the profession belittled our efforts when a few of us got together to complete the organisation of the Academy; but time has worked its levelling influences, all now is harmony and accord, and today we stand a united body with a cohesive strength, which makes membership every year more prized and more desirable.

With such an ethical standard and the perpetuation of such noble ideals and high professional motives, it is more than strange that men can be content to live and practise in this community without enjoying the protection and the assistance which affiliation with this distinguished body assures them.

We have taken in eight new members; eight members were dropped for nonpayment of dues, and two resigned. We have lost by death five esteemed colleagues,—Drs. Mynter, Lothrop, Meyer, Kraus, and Fritz. Dr. Mynter had passed well on the milestone of life, and had achieved for himself and our city a great reputation as a bold, capable and thoroughly scientific surgeon. Three of the others were young men full of promise and expectation. The academy deeply mourns the death of these five estimable gentlemen, and has passed suitable resolutions, expressing its great sorrow at their untimely separation from us. The temptation is strong for me to dwell upon the details of their noble lives, and draw from them lessons to guide and uplift us; but I must leave that for a more capable pen.

### FIBROID TUMORS.

In conforming to the custom which demands from your retiring president an address, I have had some difficulty in the choice of my subject; but, in view of the growing importance and increasing frequency of fibroid tumors of the uterus, I have chosen from this large and comprehensive field a few practical thoughts, which ten years of experience and operative work, and reading and observation have impressed upon me; and I trust they will be of interest to you.

Fibroid tumors grow in the uterus, and by reason of the contraction of its muscular fibers are pushed into different areas and localities; anatomically and clinically they are classified as subperitoneal, intramural and submucous,—according to the relative position they occupy. Sometimes they seem to grow laterally between the folds of the broad ligament, producing the so-called intraligamentary variety. The intramural and submucous ones have pain and hemorrhage as their most constant and dangerous symptoms, while the subperitoneal produces pressure upon contiguous organs, and thus interferes with the safety and well-being of their host. These tumors differ in the rapidity of their growth, and in the group of symptoms they produce, are usually recognised as benign products, and are only to be interfered with by surgical means when they interfere with the life and function of their possessors. Sometimes they attain to an enormous size, and yet their presence produces very little suffering; while again, though they be very small they may rapidly undermine the patient's health by repeated and exhaustive hemorrhages, associated with constant pain and suffering.

Three years ago, Dr. Edward J. Ill, of Newark, N. J., read a classic paper before this society, upon the indications for, and the election of, operation for uterine myomata, in which he divided the subject into three very practical divisions:

1. Those in whom life is, or might be, endangered, directly or indirectly, by the growth.

2. Those in whom health is so impaired that life becomes a burden, yet is not endangered.

3. Those in whom mental conditions suffer, from the fact that they are aware of having the tumor.

Different men will interpret these conditions in different ways, according to their instinctive desire to be radical or conservative. Many men in the profession look upon every fibroid as a very dangerous growth, and will, therefore, magnify the importance and dangers of every symptom. For instance, mere loss of blood in a woman 45 years old, if she is strong physically and can be carefully nursed, need not cause the same anxiety that it would if she were 35, because the probabilities are that the menopause will produce shrinkage in the tumor, no matter what the ultra-operative men may say to the contrary. Again, what would be a dangerous loss of blood in one person might not be so significant in another. Some women habitually lose great quantities of blood at their periods, but so quickly make it up that it seems to have very little effect upon their ordinary health and spirits. However, hemorrhage is to be considered as one of the chief dangers of fibroid tumors, particularly if the growth is of the submucous variety, and is, therefore, one of the chief indications for operative intervention.

Fibroid tumors undergo various forms of degeneration, and sometimes very suddenly take on changes which make operation imperative. Intrauterine manipulations, curetage, and electrical cauterisations, often produce septic foci in the tumor as well as necrosis and sphacelation, making a most urgent indication for operation. Pressure-producing tumors situated low down in the pelvis, which occasion great pain and inconvenience to the bowel and bladder functions, need operative interference, and particularly if bladder, ureteral and kidney complications are setting in.

In some persons these pressure symptoms are more serious and occasion more anxiety than they do in others. Marked headache, sacral neuralgia, and sciatica are often so distressing that these patients readily acquire the morphine and liquor habit for relief. Again, pedunculated growths sometimes undergo torsion by twisting of their pedicles, or produce such distress by their blows and jars upon surrounding viscera, that early interference becomes necessary.

Tubal and ovarian complications, which are so frequently associated with fibroid disease of the uterus,—such as hydro-, hemato-, and pyosalpinx, with cystic degeneration of the ovary, hematomas or abscesses of the ovary,—demand operative measures, as life is endangered by the pain and distress of these complications, and at the same time they frequently cause a local if not a general peritonitis. Tumors that undergo malignant degeneration or tumors that continue to grow after the menopause, should be quickly removed, as a mere increase in size would be strongly suggestive of malignancy. Old calcareous tumors which have been apparently innocent for years, providing they suddenly develop pain and tenderness, must be seriously considered, and upon the slightest increase of temperature must be removed, as they, too, often quickly develop pus, and are thus a great menace to life.

The presence of a myomatous growth in a pregnant uterus does not necessarily demand operative interference, as many of these women go on to term and through delivery without any trouble, because the tumor is lifted up by the growth and development of the uterus, and offers no impediment whatsoever to the incoming head and its expulsion. However, when the tumor is situated low down in the pelvic outlet, and when it remains low and will of necessity offer an obstruction to delivery, it must be removed early, and, if possible, through the vagina; but, if no active symptoms exist and none are immediately anticipated, the case, perhaps, may be left until later, when the child is viable and could be removed by Cesarean section. However, the exigencies of each case must be carefully weighed and when it seems imperative to interfere, the safety of the mother must be considered first, and her undeveloped progeny sacrificed in her interests if necessary. But every uterus with a fibroid tumor when it becomes pregnant, need not be sacrificed, even if operative interference becomes imminent, because conservative operations can often be done without causing premature labor or abortion. Emmet, in the *American Gynecological and Surgical Journal,* June, 1901, reports nine cases where abdominal myomectomy was performed in the pregnant uterus without inflicting any injury, and without causing premature expulsion of the ovum.

Supravaginal hysterectomy has such a small mortality,—not more than 5 or 6 per cent.,—that we can in good faith to ourselves and our patient's welfare, recommend it when once we are satisfied that the tumor is progressing in size and its symptoms are demanding operative treatment. There is, perhaps, more danger in being ultraconservative than in being ultraradical, and I would sooner err on the side of early operation than no operation, until the economy is so weakened by repeated floodings that self-resistance is reduced to a minimum, and death takes place from some simple intercurrent disease; or when pressure symptoms are permitted to go on until bladder, ureteral and kidney disease become serious complications. Nor are we justified in assuming that the menopause will always bring an abatement in the symptoms; but, on the contrary, a fibroid tumor, no matter if it be innocent and quiescent, must be looked upon as being always surrounded by possible harm and inherent dangers. No doubt, some continue to grow, even after the cessation of the periods, but such growths should always be looked upon with suspicion, because these tumors in old age are prone to various forms of degeneration, and occasionally to malignancy.

Two interesting cases are reported by Noble,—one being that of a woman 70 years old, who had a calcareous fibroid tumor for years, which suddenly became necrotic from a drive over a rough country road. An abscess resulted, and broke into the bowels. A subsequent operation was performed, which resulted fatally. Another, aged 67, had a calcareous tumor, which pressed upon the right ureter and caused an abscess of the right kidney. Operation was performed, and death occurred on the fourth day.

A person with an ordinary inguinal hernia, who is wearing a well-fitting truss, is comparatively safe. Yet such an individual should not get away from civilisation into the wilds of Africa, because a dangerous strangulation may set in at any time. The comparison is equally applicable to the possessors of fibroid tumors, and makes such a careful and conscientious operator as Noble, of Philadelphia, very radical in his views concerning their treatment, as so many dangers can set in infinitely greater and much more insidious than hemorrhage, which require the most skilled surgeon and without delay,—such as necrosis and sphacelation, myxomatous and cystic degeneration, with purulent and septic foci, coming on suddenly in otherwise dormant growths. Inversion of the uterus due to a fibroid is a rare condition, but would necessitate operation.

So far as any other measures are concerned for the cure of fibroids, they are simply to be condemned. Electricity once promised so much, but the dangers associated with its employment, even in careful hands, are as great as operation would be under the same favorable circumstances. So with ligation of the uterine arteries. The benefit is so transitory and so uncertain, that this procedure should never be undertaken,—at all events, only under the rarest circumstances.

The operation for the removal of the ovaries to stop the further development of the fibroid, is too uncertain. Years ago, when the dangers of hysterectomy were so great, such makeshifts were permissible; but hardly now, although there is no doubt that complete removal of the ovaries and tubes has caused diminution in the size of the tumor and a cure of the painful and dangerous symptoms, especially the hemorrhage. Therefore, in certain cases when the element of time is great, when the dangers of chloroform become manifest, and the life of the patient is in great risk, we can be content to quickly remove, thoroughly and completely, both tubes and ovaries with some expectation,—although perhaps slight,—of relief, and possible cure.

In the surgery of fibroid tumors, America may justly feel proud, since it was our surgeons who were the pioneers in this work, and who performed the first successful hysterectomy. It was Dr. Ephraim McDowell, of Kentucky, who made hysterectomy possible when he performed his first successful ovariotomy, in 1809. There was then created a necessity for the differential diagnosis of ovarian cysts and solid tumors of the uterus, because for a great many years ovariotomy was the only justifiable surgical undertaking,—in fact, the abdomen was frequently opened under a mistaken diagnosis, and finding a tumor of the uterus, the operation was abandoned and the wound closed. The history of the present operation, with its low mortality and almost perfect technic, would be incomplete without first paying tribute to the early operators in this great branch of surgery, who, under the most disadvantageous circumstances, gave us practically the operation we are advocating today.

To Clay, of Manchester, Eng., John Bellinger, of Boston, and to Burnham and Kimball, of Lowell, Mass., we must respectfully give thanks. Gilman Kimball did the first successful hysterectomy September 1, 1853, and made a correct diagnosis before the operation was undertaken. Burnham had operated successfully in June, 1853, but under a false diagnosis, having opened the abdomen for an ovarian tumor, and, finding a fibroid uterus, removed it successfully. The operations they did were essentially the supravaginal amputation most popular with us at the present time, excepting that they left the lower end of the abdominal wound open to permit the escape of the long threads which were left attached to the stump of the tumor.

With the accession of anesthesia, together with the triumphs of modern surgery, the aseptic treatment of wounds, and the employment of safe and absorbable ligature materials, refinements of technic have resulted; so that the crude operation of Kimball is now developed into one of scientific precision and exactitude. We cut off the broad ligament in sections, tie the four main vessels separately, deperitonise the uterus anteriorly and posteriorly, and use these portions of peritoneum as flaps to cover over the raw cervix, after the uterus and its tumor have been amputated. This is accomplished by a running catgut suture by which the peritoneal cavity is shut off, and an extraperitoneal disposition of the stump is effected. The abdominal wound is then permanently closed without drainage.

The first successful abdominal myomectomy was done by Washington Atlee, March 3, 1853. Thus it will be seen that American surgeons enjoy the high distinction of having performed the first successful ovariotomy, the first abdominal hysterectomy, and the first abdominal myomectomy. If we go a step further, and add to that brilliant galaxy,—McDowell, Burnham, Kimball and Atlee,—the name of Marion Sims, and later that of Emmet, our countrymen not only did the first successful operations upon the uterus for abdominal tumors, but laid the foundation of modern gynecology.

Apart from the dangers of sepsis and those accidents which are always possible when operating in the vicinity of the viscera, hemorrhage was the chief anxiety on the part of the surgeon when the stump was dropped back into the peritoneal cavity. Various devices were used to avert this danger. Elastic ligatures were applied tight about the neck of the raw cervix and left in situ; finally, the stump was pulled forwards, and in various ways was fastened to the lower end of the abdominal wound by different kinds of mechanical appliances, and thus the stump was treated extraperitoneally, so that any subsequent complication incident to it could be effectually controlled. Koeberle, by means of the serrenoeud, and his low mortality soon made the operation of hysterectomy popular and this was supplemented by the splendid results of many of his followers, chief among whom is Dr. Joseph Price, of Philadelphia, today the greatest exponent of this method of removing the uterus for fibroid tumor, and, strange as it may seem, about the only great surgeon who still clings to this practice of disposing of the stump.

It is surprising to see how slow we have been to take advantage of the knowledge which anatomy gave us so many years ago. Men had dissected the broad ligament and mapped out with exactitude the blood supply of the uterus time and again, yet it was not until 1889 that any practical application was made of this knowledge. Dr. Stimson, of New York, revolutionised the whole field of uterine surgery when he demonstrated that by tying off the uterine and two ovarian arteries complete hemostasis of the uterus could be accomplished, whereby all danger of hemorrhage from the stump is effectually overcome, and any little oozing which occasionally takes place can be further controlled by sewing over the exposed area with a catgut suture. Now we sit back and wonder why such a trivial matter was not earlier appreciated; but in doing so we must also realise that the greatest triumphs, not alone in modern surgery, but in science, inventions and mechanics, were stumbled upon, and by the simplest application of nature's laws and principles we commenced to build up a whole new universe.

Stimson's idea was quickly taken advantage of by Baer, of Philadelphia, who reported, in 1892, nine successful supravaginal amputations for fibroid tumor without a death. He paid no attention to the os. It was neither sewed up nor treated with cautery, nor any species of disinfectant or caustic. The wisdom of this procedure has been borne out by the results of the bacteriological laboratory, which have shown us that the body of the uterus contains no infecting organisms, unless they have been introduced previously by violence and other forms of traumatism; and clinically we are all able to report uninterrupted convalescence and recoveries after all kinds of complicated supravaginal operations without paying any attention to possible infection from without through the open os. In fact, most of us believe that all means directed to disinfecting the canal are quite unnecessary, and are apt to invite infection and subsequent formation of pus.

The only other operation which is employed to remove fibroid growths is complete hysterectomy, or panhysterectomy, abdominal and vaginal. The operation of total hysterectomy meets some special indications, but it is more difficult to perform, has a higher mortality, takes more time, and has increased dangers from hemorrhage; and it robs the vault of the vagina of the support which the cervix gives it. However, in certain cases it has some advantages: for example, when the cervix is the seat of marked cystic degeneration, or has a bad tear, and particularly if associated with considerable vaginal prolapse, or when drainage must be provided for. Cancer has already developed in a cervix after a supravaginal amputation, but so seldom has it occurred that we are not justified in assuming the extra risks incident to the total operation unless the cervix is lacerated or suspicious in other ways of possible malignancy. Statistics must be worth something, and, although we question them and juggle with them in various ways, they still represent the honest work of good men, and by them we must estimate the gravity and dangers of operations. The mortality after supravaginal amputation is only 5.6 per cent., while that of total extirpation in the hands of just as good men is 9.6 per cent.

Operations for fibroid through the vagina, it seems to me, must presuppose that the growth be small and the vaginal outlet large and roomy. The great objection, however, for operations per vaginam is the impossibility of conservative work, because the uterus must be destroyed; but when the abdomen is opened, one can decide as to the propriety of doing simply a myomectomy, and thus save the function and utility of the organ. Anyway, the field for operation through the vagina upon the uterus for fibroids of any size is limited, and the dangers of such operations are as great as properly directed ones per abdominem. However, occasionally submucous fibroids and polyps extruding through or presenting at the os, can often be very easily and perfectly removed through the vagina, without any great danger and with little difficulty, and such cases should always be attacked from below.

The field for abdominal myomectomy is limited, nevertheless, it is an important one; and it becomes larger the more qualified we are to master the refinements and difficulties which beset this class of operations. A young and inexperienced operator could not trust himself with the risks this work entails, but with increasing experience comes increased confidence and wisdom, and it is surprising to see how easily large tumors can be shelled out of their enclosing envelop with perfect safety, and with the possibility of leaving a healthy functionating organ. Perhaps often, in our zeal to conserve the uterus, we are taking too many risks, not only to life, but to the future well-being of the woman. To remove a great many fibroids and leave behind a mutilated uterus assumes risks which only the keenest judgment can pass upon; and even then smaller tumors may exist in the muscular wall unnoticed and go on developing to such proportions as to necessitate a total operation in the near future. Moreover, the mortality of such complicated myomectomies is high, and few women would be willing to jeopardise life simply to possess an organ which, in the end, may serve but little purpose. Of course, where a woman has a good uterus and no children, and the desire for offspring is great, and perhaps with it the possibilities of inheriting an estate, we are justified in making a greater effort, than when the social and moral conditions of the woman look more for a perfect cure than for mere relief with possible maternity.

I have been compelled to operate three times for a painful hematoma in an ovary which I had previously resected, and left in to lessen the unpleasant sequelæ which follow total oöphorectomy; and I am satisfied that, unless the indications be very strong, and the probability of future childbearing great, most of these ovaries, which are no doubt considerably diseased, should be removed and not tinkered with by so-called conservative surgery.

The foregoing table gives dates and a list of my supravaginal operations for fibroid tumors. This includes in all thirteen cases, with one death. This patient was operated upon on April 9, 1903. It was an extremely difficult operation,—a large multinodular fibroid, with its largest dimension placed antero-posteriorly in the pelvis, wedged into the hollow of the sacrum, and with great difficulty extricated. It pushed the bladder so far up and forward that the nurse did not succeed in passing a catheter into the bladder so as to thoroughly empty it before operation, according to inflexible rule. In separating the bladder, I tore a small hole into that viscus low down and posteriorly, which I quickly and carefully sewed up. I felt reasonably certain that it would hold, but evidently it did not, because the patient died on the third day, and the undertaker told me that when he used the trocar for embalming purposes a urinous-smelling fluid came out of the peritoneal cavity. A postmortem was denied us. Had I done a complete hysterectomy and left the vagina open and provided a gauze drain, I feel satisfied that my patient would have made a good recovery. About four hours after the operation, I withdrew with a hard catheter over a pint of urine that contained pus,—evidently residual urine in a sacculated bladder, which the woman had failed to pass with her efforts at urination, and which the nurse had failed to reach with a soft catheter. This distension of the bladder no doubt contributed to the little tear I made in separating it from the uterus.

I have also done a number of myomectomies, vaginal and abdominal, and a number of total hysterectomies, vaginal and abdominal, for various forms of fibroid disease, but I prefer the supravaginal amputation as given to us by Baer, when the abdomen must be opened and the uterus sacrificed, and occasionally I have modified it by taking advantage of the suggestions of Kelly, Goffe, and many other recent operators, when some special indication seemed to exist for their employment.

493 Delaware Avenue.

---

Tubercular Peritonitis.—The best results follow a median incision below the umbilicus, free evacuation of fluid and immediate closure of the abdomen.—*Denver Med. Times.*

